# Overview of approved COVID-19 vaccines in the EU, recommendations for use in Sweden and vaccine uptake over time: Report from the Swedish Medical Products Agency and the Public Health Agency of Sweden

**DOI:** 10.48101/ujms.v130.12937

**Published:** 2025-10-16

**Authors:** Björn Zethelius, Johanna Rubin, Nicklas Pihlström, Ulla Wändel Liminga, Helena Back, Bernice Aronsson, Anders Tegnell, Ulrika Marking, Jonas F. Ludvigsson, Sören Andersson, Rickard Ljung

**Affiliations:** a Use and Information Division, Swedish Medical Products Agency, Uppsala, Sweden; b Department of Public Health, Clinical Geriatrics, Uppsala University, Uppsala, Sweden; c Public Health Agency of Sweden, Stockholm, Sweden; d Division of Paediatrics, Department of Clinical Science, Technology and Intervention, Karolinska Institutet, Stockholm, Sweden; e Licensing Division, Swedish Medical Products Agency, Uppsala, Sweden; f Department of Clinical Sciences, Danderyd Hospital, Stockholm, Sweden; g Department of Medical Epidemiology and Biostatistics, Karolinska Institutet, Solna, Sweden; h Department of Pediatrics, Örebro University Hospital, Sweden; i Department of Medicine, Columbia University College of Physicians and Surgeons, New York, NY, USA; j Institute of Environmental Medicine, Karolinska Institutet, Stockholm, Sweden

**Keywords:** COVID-19 vaccines, safety, adverse drug reactions, epidemiology, public health

## Abstract

**Objective:**

The aim of this review is to describe the regulatory background of the COVID-19 vaccines, the national recommendations for use issued and vaccine uptake in Sweden. It includes an overview of licensing and relevant safety aspects identified by the European Medicines Agency (EMA) and the national vaccination plan issued by the Public Health Agency (PHA) of Sweden.

**Materials and methods:**

Information on dates of licensing and safety aspects of importance identified by EMA published on its website, was compiled and presented in a chronological order. National recommendations on COVID-19-vaccination and vaccinations-data on uptake and coverage using the national-vaccine-register are presented.

**Results:**

COVID-19 vaccines development, assessments using rolling review and licensing of the covid-19 vaccines was done in 2020 during less than a year. Large-scale production was implemented. Monthly safety reviews performed by the EMA identified risk for thrombosis with thrombocytopenia syndrome with adenoviral vaccines and myocarditis for mRNA vaccines which led to restrictions in national recommendations for specified groups.

National vaccinations were launched in a phased manner during 2021. Persons of high age, risk groups and nursing home personnel were prioritised during primary vaccinations and for initial boosters. In the Swedish population, 85% recieved at least on vaccine dose from the age of 12. At least two doses were recieved by 81% from age 18 and 95% from age 80.

**Conclusion:**

Recommendations for national use adhered to relevant adverse drug reactions identified. The vaccine coverage was high. Timelines presented should be considered in follow-up studies of COVID-19-vaccines to manage possible selection bias and confounding.

## Introduction

Vaccines play a crucial role in curbing the spread of a pandemic virus. The COVID-19 pandemic hit the world in the beginning of 2020 and intense and rapid vaccine development was launched. Such rapid development was enabled by the high public health need, by novel platform technologies, especially the mRNA technology and by the possibility to undertake large clinical trials covering immunogenicity, efficacy and safety within very short time frames, because of the high COVID-19 case numbers (1). Furthermore, optimal use of procedural systems for approval of medicines with unmet medical needs in the European Union (EU) contributed to rapid approval of the first COVID-19 vaccine in the EU in December 2020. Three additional COVID-19 vaccines were then approved between January and March 2021.

Taken together, the time from the first case identified in Europe to initiating vaccination campaigns was roughly 1 year.

The new COVID-19 vaccines were reviewed by the Committee for Human Medical Products (CHMP), at the European Medicines Agency (EMA) (2, 3) in rolling review processes until sufficient data on quality, efficacy and safety had been obtained to make a positive conclusion on the benefit-risk balance, within conditional marketing authorisations. The Swedish Medical Products Agency (SMPA) is one of the partners in the regulatory network (4) and acted as rapporteur of the first approved vaccine to be used within the EU. Pharmacovigilance activities were extensive and were carried out by the national relevant authorities, the EMA and the Pharmacovigilance Risk Assessment Committee (PRAC) at the EMA (5).

Access to vaccines in the EU was made possible by the partnership of the EU co-operation under a procurement contract for purchases of vaccines against COVID-19. The Public Health Agency of Sweden (PHA) was commissioned by the Swedish government to create a national vaccination plan (6). It was implemented as a four-phased vaccine distribution plan for primary vaccinations, initially prioritising vaccination of those at highest risk of COVID-19 complications and those at high risk of exposure and transmission. Vaccinations against COVID-19 in Sweden started on 27 December 2020. The initial goal was to offer at least one dose to all adults in Sweden before July 2021.

The aim of this review is to describe and highlight the timelines and chronology of CHMP opinions and subsequent decisions from the European Commission and the risks identified by the PRAC which had an impact on the PHA national recommendations on the use of vaccines. In the review, we describe the recommendations for primary vaccinations from the start of the campaign as well as for onward booster doses. Furthermore, data on vaccination uptake and coverage over time from start of the campaign until 31 of December 2024 in the Swedish population will be presented.

## Regulatory background

Scientific evaluation, supervision and safety monitoring of medicines for human use in the EU are generally performed within the EU regulatory network, consisting of the EMA in close cooperation with EU institutions and member states (2). The SMPA is one active partner in this network (4). Marketing authorisation applications of many new vaccines to be used in EU are obliged to be assessed via the EMA within the centralised procedure before granting marketing authorisation (3). Safety surveillance in the EU is mandatory and the PRAC is reviewing the safety of medicines at their monthly meetings (5). The PHA is the Swedish national authority recommending on the use of vaccines in Sweden (6).

### EMA marketing authorisations

Four vaccines out of the eight approved by the EMA and the EU-commission which are listed in the Appendix Table A1 were used in Sweden. These were two mRNA vaccines: Comirnaty (BNT162b2, Pfizer BioNTech) and Spikevax (mRNA-1273, Moderna), one viral vector vaccine Vaxzevria (ChAdOx1 nCoV-19, Oxford-AstraZeneca), and one protein subunit vaccine Nuvaxovid (SARS-CoV-2 subunit spike-protein by NOVAVAX). The dates for marketing approval of respective vaccines in specified age and risk groups are presented in the Appendix Table A1. Large-scale productions were implemented by the pharmaceutical industry, not further discussed here.

### EMA pharmacovigilance activities

For the COVID-19 vaccines, pharmacovigilance activities were extensive, including signal detection, monthly safety reviews and the conduct of post authorisation safety studies. Assessments were performed via the competent national authorities together with the EMA. They were subsequently discussed in the PRAC and risk communication activities were coordinated. Two important adverse drug reactions (ADR) were identified in 2021 for viral vector and mRNA vaccines, respectively, that affected national vaccination recommendations in Sweden. They were thrombosis with thrombocytopenia syndrome (TTS) and myocarditis and pericarditis, respectively. Both these important safety issues will be discussed further in the text. A direct health care professional communication (DHPC) to relevant target groups of health care professionals was sent for these two important risks identified for necessary actions needed to be taken; see Appendix Table A2 A-B and A4 A-B including links to the EMA website.

Further, recommendations on use in pregnancy are presented with more details in the Appendix Table A4 A-B, panel B. Pregnant women were not included in the vaccine trials for precautionary reasons and thus clinical trial data were lacking.

## National vaccination plan

The PHA was commissioned by the Swedish government (6) to develop a national vaccination plan. It was conducted in a phased manner, for primary vaccinations against COVID-19, and further recommendations on booster doses were made during the pandemic as described in Figure 1. A condensed overview of the most important recommendations is presented in Table 1. In the Appendix Table A5, all decisions and recommendations by the PHA are listed in chronological order. Over time a change in parlance has occurred and from 22 June 2023 the wording ‘a single dose’ is used in EMA posology instead of ‘primary vaccination’ or ‘booster’ dose. However, here we use the term booster throughout the text. The term refill is used in Figure 1 (PHA).

**Figure 1A:**
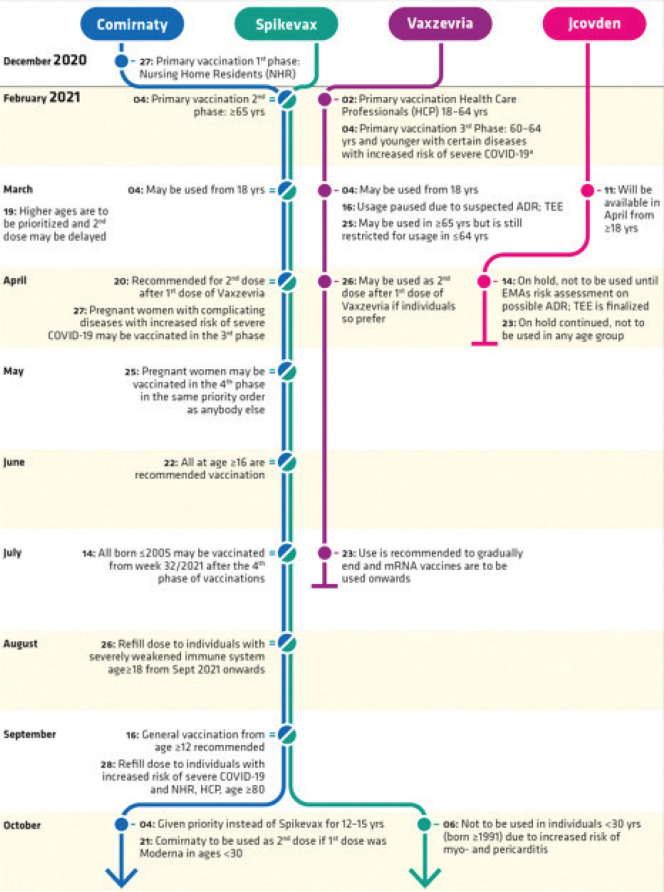
Flowchart over recommendations issued by the Public Health Agency of Sweden on the use of COVID-19 vaccines in Sweden from 27 December 2021 throughout 2024, including the winter season 2024 to 2025.

**Figure 1B:**
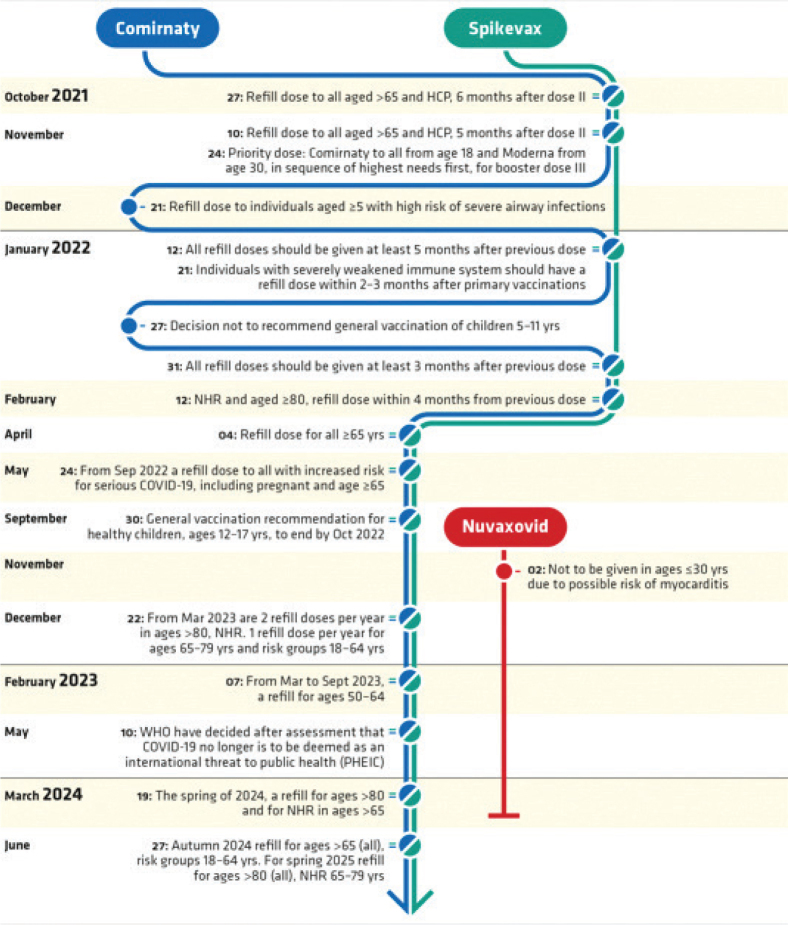
Flowchart over recommendations issued by the Public Health Agency of Sweden on the use of COVID-19 vaccines in Sweden from 27 December 2021 throughout 2024, including the winter season 2024 to 2025.

**Table 1:** Timetable for main national general recommendations in Sweden issued by the Swedish Public Health Agency by age group for vaccination with covid-19 vaccinations, listed by dose number, in Sweden 2020–2024.

Age (years) / Dose	80+	65–79	18–64	≥16	≥12
I and II*	27 Dec 2020	04 Feb 2021	04 Mar 2021**	22 Jun 2021†††	06 Sep 2021†††
III***	28 Sep 2021	27 Oct 2021	24 Nov 2021**	-	-
IV†	14 Feb 2022	04 Apr 2022	50 +, 07 Feb 2023 to 09 Sep 2023	-	-
V†	01 Sep 2022	01 Sep 2022	-	-	-
VI††	01 Mar 2023	01 Sep 2023	-	-	-
VII	01 Sep 2023	01 Sep 2024	-	-	-
VIII	01 Apr 2024	-	-	-	-
IX	01 Sep 2024	-	-	-	-

* The initial recommendation included vaccination with two doses with an interval of 4 to 6 weeks. The interval was adjusted temporarily at different times during spring 2021 reflecting vaccine supply. Doses I and II were implemented following a priority list prioritising nursing home residents and elderly requiring homecare.

** Stepwise access from 64 down to 18 years of age.

*** Interval between dose II and dose III was successively shortened from 6 to 3 months during spring 2022 due to the spread of the virus variant omicron.

† Recommendations on further booster doses could be applied on risk groups from dose number V.

†† From spring 2023, dose number was no longer communicated in recommendations. It was replaced with recommendations on one or two doses yearly, depending on the risk group a person belonged to.

††† Two doses as a general recommendation for children <18 years old ended 31 October 2022.

### The phased national vaccination plan.

The aim was to offer vaccination to those in greatest need of protection against severe COVID-19. The PHA recommended primary vaccinations to be performed according to a priority listing in four phases (6). It included recommendations on when specified groups would be offered vaccination.

– The first phase recommendations included elderly individuals living in long-term residential care facilities or in at-home care settings including family members living with them and the staff at these facilities. It started on 27 December 2020.
– The second phase included individuals aged 70 years and older, or immunocompromised persons. It started on 04 February 2021.
– The third phase included other adults in predefined risk groups of severe COVID-19 as well as those with difficulties following advice on infection control measures, such as individuals with cognitive or mental functional impairments. It started on 20 April 2021.
– The fourth phase (last phase) included the remaining adult population. It started on 25 May 2021.

Booster doses were recommended from 26 August 2021, initially for individuals with severe immunodeficiency and then in a phased manner beginning with those at greatest risk of severe COVID-19 from 28 September 2021. Further recommendations on booster doses from 12 January 2022 and onwards, included different time periods since last dose for specified groups.

### Primary vaccinations

The first two doses, that is, primary vaccinations, were made available as described earlier in the text and to progressively younger age groups over time. The initial goal was to offer at least one dose before July 2021 to all adults in Sweden, but this goal had to be postponed to late September because of lack of vaccine deliveries and the early identification of the thrombosis with TTS that lead to restricted use of Vaxzevria and later withdrawal from the market.

Also contributing to postponed goals above were varying availability of vaccines because of short time frames for vaccine deliveries within the EU, the short shelf life’ on delivered vaccines and varying amounts of vaccine per shipment to Sweden. Thus, national priorities and national recommendations on timelines had to be repeatedly adapted and changed accordingly. For recommendations of use in children and in pregnancy, please see the Appendix.

### Booster doses

A third dose was first offered in September 2021 to individuals in older age groups at a given time interval after their second dose. Over time, the third dose was offered to younger age groups and with shorter dose intervals. A third dose was not offered to those under 18 years of age unless there were special considerations. Further, in February 2022, a fourth dose was made available to those aged over 80, to individuals living in long-term care facilities or in at-home care settings including family members living with them, to the staff at these facilities and to those with severe immunodeficiency. This was extended to those over 65 years of age in April 2022. In September 2022, a fourth dose was made available to those over 18 years; however, availability varied between regions. Exceptions could be made under specific circumstances, that is, earlier vaccination for immunocompromised individuals or for those taking care of vulnerable individuals. Furthermore, in September 2022 a fifth dose was made available to those with increased risk for serious COVID-19 including pregnant women and those over 65 years.

– In June 2023, the updated Summary of Products Characteristics (SmPC) for the mRNA vaccines stated yearly single doses for risk groups, and two-yearly single doses for high-risk groups, moving away from the previously used concept of primary vaccinations doses and booster doses.
– In March 2023, individuals aged 80 years and older were recommended two booster doses per year and those aged 65–79 years one booster dose per year. Further, healthy individuals aged 50–64 years old who had been vaccinated earlier were not recommended a booster before the winter season 2023 to 2024. However, previously unvaccinated individuals 50–64 years were recommended a single dose.
– In March 2024, those above 80 years and those above 65 years in long-term care facilities were recommended another booster.
– On 10 May 2024, the WHO declared that COVID-19 no longer was a Public Health Emergency of International Concern and thus, in June 2024, PHA recommendations were set that all aged 65 and older, and those aged 18 and older at risk for severe COVID-19-disease should be offered vaccination for the upcoming season 2024 to 2025.

## Regional vaccination activities

In Sweden, there is regional self-government according to the Swedish constitution (7). The PHA issues recommendations but cannot decide in detail how the 21 self-governing regions should perform the vaccinations. Thus, the regional authorities decide on the actual order of priorities and procedures to adjust their implementation to logistic and specific local circumstances. Priorities have somewhat varied in the different regions, mostly within the recommended ‘four-phases’ priorities.

Possible lack of national coherency was counteracted by governmental financial support to the regions if certain coverage goals, that is, milestones, were met at predefined deadlines. Follow-up of vaccination coverage (8) and types of vaccine used showed very small regional differences.

## Detection of circulating COVID-19 variants

**Figure A1:**
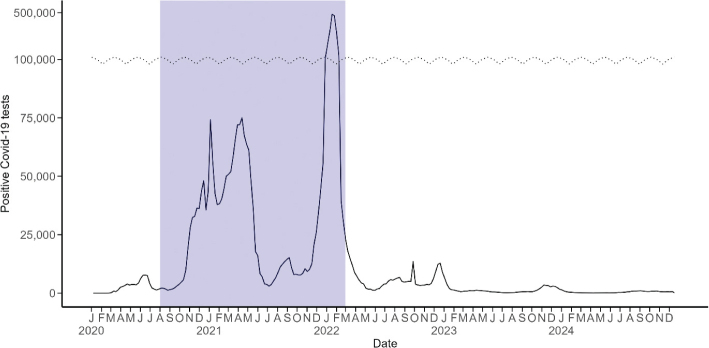
Weekly number of positive SARS-CoV-2-tests from January 2021 to 31 December 2024. The Y-axis is compressed between values 100.000 and 500.000 to give more detail to values below 100.000. There is a marked peak of testing in the first quarter of 2021. The period of freely available testing from 01 August 2020 to 28 February 2022 is given in the figure as the coloured square.

**Figure A2:**
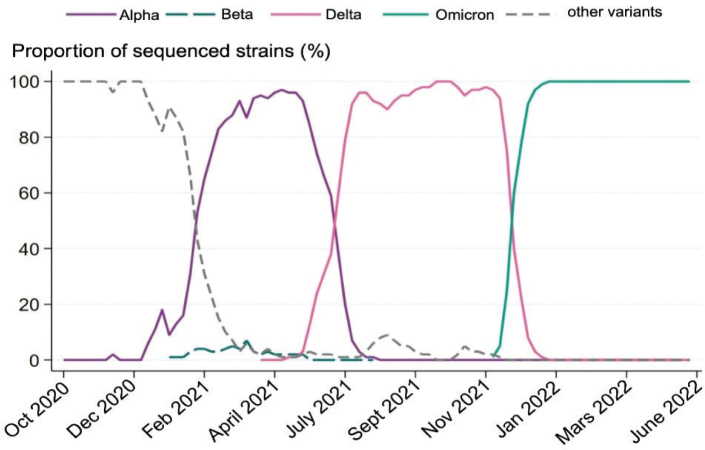
Proportions of sequenced strains of SARS-CoV-2 in positive tests over time from 2020 to 2022, that is, alpha, beta, delta and the omicron variants.

Testing for COVID-19 (mainly performed by reverse transcriptase–polymerase chain reaction (PCR) assays or immune-based antigen tests) was performed at regional laboratories. Positive SARS-CoV-2-tests were reported to be registered in the SmiNet database, held by the PHA. In the Appendix, Figure A1 the weekly number of positive SARS-CoV-2-tests from January 2020 to 31 December 2024 are shown and in Appendix Figure A2 the proportions of sequenced strains of SARS-CoV-2 in positive tests over time from 2020 to 2022, that is, alpha, beta, delta and omicron are presented. A continuous monitoring of variants of concern is performed by the European Centre of Disease Prevention and Control and presented in monthly updated listings (9).

## Follow up of vaccine safety and vaccine effectiveness

The SMPA launched according to governmental assignment, the COVID-19 VACcination register SAFEty study in Sweden (CoVacSafe-SE) platform (10) for epidemiological surveillance to detect and characterise suspected adverse effects, vaccine effectiveness and vaccination coverage of COVID-19 vaccines in Sweden. It includes all individuals 12 years or older in Sweden in 2021 and onwards. Data obtained from several national registers to the platform are regularly updated: Vaccine doses and positive COVID-19 tests are updated weekly (10).

### Ethics

The CoVacSafe-SE platform was approved by the Swedish Ethical Review Authority (2020-06859 and 2021-02186).

## National vaccination uptake

Using the national vaccination register including information on numbers and dates of individual doses, the uptake and national coverage was calculated. This is presented in the following figures. Approximately six times more individuals were vaccinated with Comirnaty than with Spikevax because of higher availability of the former.

**Figure 2:**
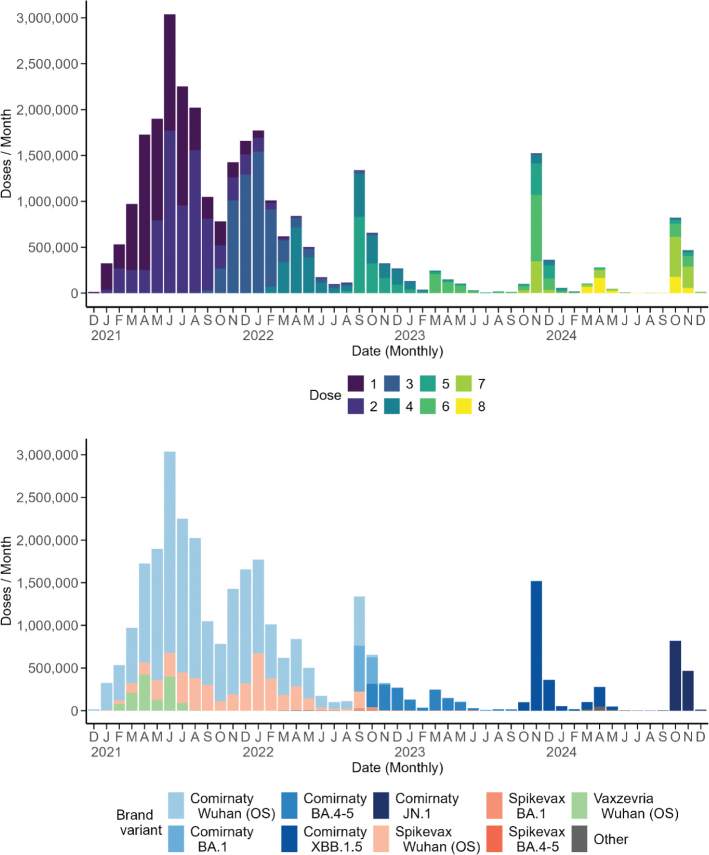
Monthly uptake on doses, number one to number eight, colour coded, in the Swedish population, age 12 years and above from 27 December 2021 to 31 December 2024 in the upper panel and the monthly uptake by vaccine variants in the lower panel.

In Figure 2, the monthly uptake by dose number one to eight from 27 Dec 2020 to 31 December 2024 is presented in the upper panel. The primary vaccinations, that is, doses one and two were performed from January 2021, peaking in June and fading out in January 2022. Administration of the first boosters, that is, dose three started in October 2021, peaking in January 2022 and fading out during the summer months of 2022. Second boosters, that is, dose four started in February 2022, peaking in April and fading out in the late summer of 2022. Later boosters, dose five peaked in September 2022; dose six in May and in November 2023; dose seven peaked in November 2023 and in October and November 2024. Dose eight peaked in August and November 2024. All in all, these peaks reflect the time dynamics of vaccination activities in response to updated national recommendations.

**Figure 3:**
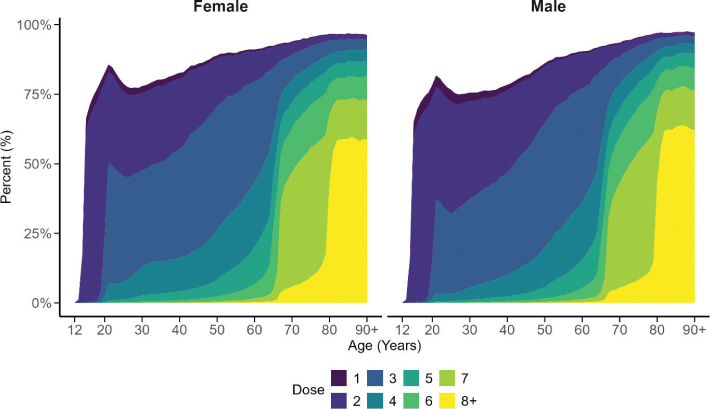
Total uptake of vaccine doses number one to number eight and above combined, colour coded on 31 December 2024 in females (Left panel) and males (Right panel) aged 12 -100 years as percent vaccinated per 1-year stratum from 27 December 2020 and forward for those still alive on 31 December 2024.

The monthly uptake by vaccine variants is shown in Figure 2, lower panel. Only the Comirnaty BA.4-5, XBB.1.5 and JN.1-variants were administered, and in this sequence, from 2023 and onwards. Total uptake of vaccine doses number one to number eight and above combined is presented from 27 December 2020 and onwards for those aged 12 years and older and still alive on 31 December 2024 in Figure 3. No clear difference between women and men was observed.

Dose intervals, for example, median 42 days between dose 1 and dose 2 and 189 days between dose 2 and dose 3, are presented, for all dose intervals from dose 1 to dose 10, in the Appendix, Table A6.

### Time-dynamic animation of vaccine coverage in Sweden

**Figure 4:**
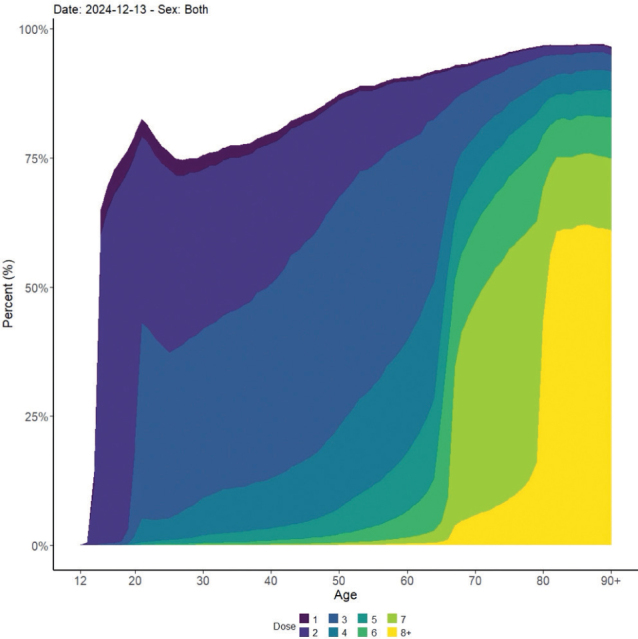
Time-dynamic animation from 01 January 2020 until 31 December 2024 of vaccine uptake in the Swedish population age 12 years and above and still alive on 31 December 2024. The speed in the animation is 1 week per second. The doses are colour coded. Use the link below to access the presentation: https://ujms.net/index.php/ujms/article/view/12937/19909.

Uptake of vaccine over time is illustrated in a time-dynamic animation in Figure 4 including the Swedish population (age 12 years and above and still alive on 31 December 2024) from January 2020 to 2024. The speed in the animation is 1 week per second and doses are colour coded illustrating the sequence over time. Use the link below to access the presentation: https://ujms.net/index.php/ujms/article/view/12937/19909

## Discussion

Here we present a chronological compilation of dates for marketing authorisations of the COVID-19 vaccines used in Sweden and the variations in their indications, as well as dates of important safety concerns communicated and national recommendations on Covid-19 vaccine use during years 2021–2024. This compiled information is important when designing and performing non-interventional COVID-19 vaccine studies, Further, even if it is beside the scientific scope, such compiled information on dates when relevant decisions were made, is essential when evaluating the pandemic management. A discussion on possible advantages and disadvantages of Sweden’s national vaccine recommendations as compared to our neighbouring Nordic countries with similar health care systems is beyond the scope of this paper.

### European marketing authorisations and pharmacovigilance

Rolling review submissions to the EMA started with preclinical data evaluations in October 2020 and subsequently, the data from clinical studies and product quality were provided. This allowed European regulators early access to available data needed to support approval and thus allowed for prompt and progressive review of data almost in real time. This enabled accelerated assessments and approval of the first COVID-19 vaccines as soon as sufficient data were available. Also, vaccines adapted to the changing virus biology became available with time. Likewise, pharmacovigilance activities were substantial, including intensified signal detection work and monthly safety updates on emerging safety data, allowing for early detection of new risks.

### National recommendations – Phased plan

In Sweden, a phased plan for primary vaccinations was chosen prioritising the most vulnerable individuals and health care personnel. World-wide, health care personnel were prioritised to varying extents (11). Initially, younger individuals and children were not included in the prioritisation in Sweden, the latter in accordance with WHO guidance (12). Pregnant women were not initially prioritised because of lack of clinical trial data. A recent study compared disparities in vaccine policies and its effectiveness in three European countries and found differences, even if the same start date for vaccinations (13) was applied and that vaccination supply was coordinated at the EU-level. In the United States, guidance was made at state level and vaccinations started in a phased manner on 14 December 2020 (14).

### Recommendations on dose intervals in primary vaccinations

The interval between doses one and two was somewhat longer than recommended in the posology of the SmPC as it was decided by the PHA to aim for a broader uptake of the first dose within a shorter time frame rather than to fulfil the SmPC posology. This approach: ‘One dose to as many as possible’, chosen in Sweden was similar to the approaches in the other Nordic countries. Given the limited numbers of vaccine doses available in the early phases of the vaccination campaign, one dose to everyone was prioritised ahead of two doses to some groups, that is, this was made to gain a partial, but predicted crucial, immunity for as many as possible before continuing with the second dose. Thus, aiming for the greater good this pragmatic approach did not always comply with the SmPC.

### Recommendations on booster doses, later called single doses

Vaccine effectiveness wanes over time. Thus, COVID-19 vaccine boosters were needed. As strains of SARS-CoV-2 changed over time, modified vaccines were developed and used sequentially with new variants replacing previous, according to updated vaccination recommendations. For vaccine effectiveness studies, it is thus important to consider previous vaccination schemes, dates for recommendations and dates for most prominent virus strains (15, 16). In Sweden, more frequent booster doses were recommended for the elderly and those at greater risk for severe COVID-19; see Appendix Table A5.

Although countries within the EU established national criteria, differences were later not that large, as policies converged with time (17).

### Adverse drug reactions affecting national vaccination recommendations

Two ADRs affected national vaccination recommendations in Sweden during the spring and the summer to autumn of 2021. A signal review was started by PRAC on 12 March 2021 because of reports of embolic and thrombotic events from Vaxzevria, that is, an important potential risk as defined by the EMA (18) for this vaccine. On 18 March 2021, the PRAC concluded that thrombosis with thrombocytopenia was a rare adverse reaction to this vaccine. This condition was later renamed to thrombosis with thrombocytopenia syndrome (TTS). A DHPC was published on the EMA website on 24 March 2021 (19). The PHA recommended on 16 March 2021 that Vaxzevria should not be used in individuals younger than 65 years of age. Initially, Vaxzevria was recommended for use among those aged 64 years and below until more data became available because of less robust immunogenicity data in ages above 55 years when first approved. Vaxzevria, was still to be used in the elderly until July when it was recommended not to be used at all in Sweden. It was later withdrawn from the market. Thus, this serious safety concern with Vaxzevria raised a need to prioritise the use of mRNA vaccines instead. In contrast, no restrictions were made for the use of Vaxzevria in the UK. For JCovden, also a viral vector vaccine, a similar signal review was started in April 2021, and PRAC concluded later in the same month with TTS being a rare risk for JCovden also. The PHA never introduced this vaccine in the national vaccination campaign. For both vaccines, DHPCs (19, 20) were sent out to a wide range of health care professionals.

In June 2021, the PRAC started a signal review of myocarditis and pericarditis for both mRNA vaccines Comirnaty and Spikevax, based on spontaneously reported cases. In July 2021, the PRAC concluded that myocarditis and pericarditis are very rare adverse reactions to both vaccines (21). Also in this case, a DHPC (22) was sent out to a wide range of health care professionals. On 06 October 2021, based on data from Denmark, Finland, Norway and Sweden in a joint Nordic study presented to the Public Health Agencies in the Nordic countries, the use of the mRNA vaccine Spikevax was restricted in ages under 30 years. This was because of an observed higher absolute risk for myocarditis and pericarditis compared to Comirnaty (23). Later, in December 2021, EMA communicated the findings from this and other epidemiological studies in an updated signal review of myocarditis and pericarditis (24).

Additional safety concerns with the vaccines in use were reviewed but no substantial new risks, in need for specific communication, were identified from PRAC assessments. The regulatory agencies’ safety assessment and risk communications were within days considered for national PHA recommendations. National recommendations were communicated to the regional authorities performing the vaccinations and adherence of the regional authorities to the national recommendations was deemed high.

### National recommendations on vaccinations during pregnancy

Vaccinations for pregnant women were initially not recommended because of lack of clinical trial data at first assessment for marketing approval. With time, knowledge about the risk for severe disease during pregnancy increased and reassuring safety data related to use of COVID-19 vaccines during pregnancy from other countries became available (25, 26). Thus, in the spring of 2021 the PHA recommended vaccinations during later parts of pregnancy. The EMA undertook a detailed review of available data during 2021, see Appendix, Table A4 A-B, panel B. The growing evidence indicated that Comirnaty and Spikevax can be safely used during pregnancy and breastfeeding and thus, the SmPC was updated accordingly in February 2022 (27). Later, the Comirnaty SmPC was updated in November 2024 with further support from a phase 2/3 study in healthy pregnant women aged 18 years and above (28).

### National recommendations for vaccinations in children

Comirnaty was first approved from age 16 years in December 2020 and Spikevax from age 18 years in January 2021. Later, when data became available, these vaccines were approved for use from age 12 years in May and July 2021, respectively. However, recommendations for vaccine use in children 12 years and above in Sweden were first issued by the PHA in September 2021. Caution was considered warranted in the youngest with lowest risk for severe outcome of a corona virus infection. The recommendations were aiming to avoid possible vaccination adverse reactions, that is, myocarditis and pericarditis. Thus, from November 2021, only Comirnaty was recommended for use below 31 years of age. The PHA recommended general immunisation of children from 12 years of age until 31 October 2022. There was never a general recommendation for children below 12 years of age, nor was there a recommendation of a third dose to those aged 12–15 years in Sweden.

### Vaccine uptake and coverage

Reporting of COVID-19 vaccine doses administered was made mandatory to the national vaccination register at the PHA from the start of the vaccination campaign as of a change in the legislation (29); ‘Act amending the Act’ (2012:453) on registers of national vaccination programmes. Number of vaccine doses reported were updated daily and publicly displayed on the PHA webpage. This readily available continuous feedback from the PHA to the vaccinators and health care regions supported decision making on several levels and effective steering of the vaccination campaign process. Overall, uptake for primary vaccinations was high during 2021 and faded out during 2022. This was followed by high uptake of third and fourth doses in prioritised groups during 2022. In 2023 and 2024, mainly additional single doses in specified age and risk groups were administered.

Coverage for primary vaccinations with one dose was 85% and for two doses 81% in adults. In the age group 80 years and above with the highest risk of developing severe COVID-19, the coverage was around 95% for the first two doses given. Coverage of primary vaccinations may thus be deemed high on the population level, both in Sweden and in other European countries (30). Overall, coverage should be seen in the view of possible challenges in reaching out to the whole population with the COVID-19 vaccination campaign in Sweden. A poll conducted by the PHA in April–May 2021 showed that around nine of 10 Swedes were willing to get vaccinated (31); however, it was observed that foreign-born Swedes were less inclined to get vaccinated. Further, lower vaccination uptake was observed to be associated with lower income and education level, respectively (31).

### Relevance of strain-adapted vaccines against SARS-CoV-2

Continuous follow up of circulating virus variants was important for assessment of relevance of current vaccine targets. The Swedish situation did not differ in any important way from the situation in other countries (9) and adaptation of the vaccines to major circulating COVID-19 variants was relevant and the vaccines used were in general of the latest strain adaptation. The Omicron variant of the virus was dominating in Sweden from 21 December 2021 and because of high transmissibility of this variant there was within a few weeks a rapid spread of the virus in the Swedish population, Appendix Figure A1. In parallel with this change in transmissibility the numbers of severely ill patients because of COVID-19 started to decrease. Reasons for that could be that the omicron variant of the virus was less pathogenic, although highly transmissible and because of gradually increasing immunity in the population.

### Viral biology and future perspectives on follow-up studies

Future variations of COVID-19 vaccine composition because of upcoming changes in viral biology with new mutations are foreseen to be continued as well as recommendations on yearly seasonal vaccinations against COVID-19. For studies on effectiveness, safety and coverage of these vaccines in the EU using vaccination register data, it is of importance to consider the dates for country-specific recommendations on vaccine use, that is, the differential roll out of vaccines, dates on when approvals occurred and when serious safety signals were detected and communicated as well as the temporal spread of different virus variants. The latter is more difficult to control for, as data on which virus variant that was dominating during a certain period, that is, positive SARS-CoV-2-tests, is affected by the availability in different countries of testing over time. In essence, the paper highlights the need of taking important information and time points of regulatory decisions and national recommendations on use of the COVID-19 vaccines into consideration when selecting and defining a study population of interest pending a research question posed. Further, information presented here is needed to consider also when analysing and managing time dynamics, selection bias and confounders in longitudinal follow-up studies of COVID-19-vaccines, for example, that patients vaccinated before the start date of a national recommendation probably to a higher extent are risk group patients (32). This paper covers all dates for future assessments of the vaccination strategy.

### Lessons learned for better future preparedness

Reasons for the short approval processes during COVID-19 pandemic were: The high public health need because of rapid spread of the disease, the availability of novel platform technologies and an optimised use of procedural systems for development of emergency vaccines. Further, large-scale, so-called seamless clinical trial designs were adopted where the phases of vaccine development were formed into one single protocol, adapted as needed over time and approved of by regulatory agencies that contributed to shortened timelines of large-scale trials performed. Additionally, as the COVID-19 case numbers were considerably high, the clinical trial efficacy endpoints were met within short time frames. This combined with the EMAs rapid rolling review assessments of data obtained instead of the standard centralised procedure timelines lined out in the EU-legislation (2, 3) sped up time for approval considerably. Thus, fast processes on development, regulatory assessments and political decision making underpinned public health recommendations on vaccine use in a crisis that was handled fast at the EU level and in the countries within the EU during the evolving pandemic health crisis.

Consequently, several steps have been taken in the EU to meet different future cross-border health crises via the Health Emergency Preparedness Response (HERA) initiative by the EU-commission (33). To overcome future pandemic outbreaks, requiring the manufacture to provide several billion doses of vaccines within months there are several needs to be met: Viral biology surveillance; Fast tracked regulatory processes; A rapid-response industry using new manufacturing platform technologies and preparedness for large scale production; Decentralised manufacturing, production and distribution processes; Streamlined public health measures and recommendations by the WHO and national health agencies and sufficient financial support. Taken together, efforts during the COVID-19 pandemic have increased awareness and preparedness for rapid response to challenging future pandemics potentially involving other viruses. Potential solutions for providing vaccines for pandemic response at an unprecedented scale and rate is always, at least in part including the handling of the unknown. For the follow-up of vaccine coverage and safety it is important to have registers up and running from the start of a vaccine campaign.

In May 2025 the World Health Assembly adopted a historic Pandemic Agreement to make the world more equitable and safer from future pandemics (34). Thus, collaboration is the key factor for the aspects discussed earlier in the text to meet future pandemic challenges which also apply on the national level for interactions between agencies, health care providers and vaccinators.

## Concluding remarks

The current paper presents comprehensive information in chronological order on dates of approvals and variations of the COVID-19 vaccines in the EU, on safety updates communicated from EMA, as well as dates on which and when different groups in Sweden were recommended vaccination. It presents when and what vaccines and variants thereof were available and to whom, considering subjects at greatest need for protection as well as emerging safety concerns for certain groups. Further, it presents when Vaxzevria was no longer recommended for use in Sweden during primary vaccinations, the concurrent increased need for the mRNA vaccines and the later dominating use of Comirnaty in the Swedish population. This in-depth review highlights the fact that regulatory decisions, national recommendations and vaccine uptake should be considered cornerstones when performing non-interventional studies using COVID-19 vaccination exposure data. The lessons learned from the pandemic have increased preparedness in the EU countries and in the world-wide community for future cross-border health crises management. Establishment of the vaccine register at the national level before the start of the vaccine campaign was instrumental for follow up.

## ORCID

Björn Zethelius https://orcid.org/0000-0002-1738-0834

Johanna Rubin https://orcid.org/0000-0003-1541-0064

Nicklas Pihlström https://orcid.org/0000-0001-6812-0971

Ulla Wändel Liminga https://orcid.org/0009-0004-3703-7867

Helena Back https://orcid.org/0009-0009-7444-3975

Bernice Aronsson https://orcid.org/0009-0003-7326-9879

Anders Tegnell https://orcid.org/0009-0005-8319-721X

Ulrika Marking https://orcid.org/0000-0002-5580-4191

Sören Andersson https://orcid.org/0000-0002-4522-3078

Jonas F. Ludvigsson https://orcid.org/0000-0003-1024-5602

Rickard Ljung https://orcid.org/0000-0002-0654-4530

## Transparency

The authors affirm that this manuscript is an honest, accurate and transparent account of the compiled data being reported; that no important aspects of the data have been omitted.

The manuscript has been reviewed and approved by the SMPA scientific quality assurance board.

## Data sharing

The aggregated data presented in this study are available upon request. The origin of the data is the Swedish national vaccination register held by the Public Health Agency.

## Dissemination to participants and related patient and public communities

Patients were anonymised in the utilised data sources; and therefore, direct dissemination to them is not possible.

## Appendix

**Timelines of approval and safety announcements of COVID-19 vaccines from the European Medicines Agency and national recommendations for use in Sweden**

**Report from the Swedish Medical Products Agency and the Public Health Agency of Sweden**

**Table A1:** The four vaccines used in Sweden for immunisation against SARS-CoV-2 virus infection and dates for approved marketing authorisations and extensions of indication.

COVID-19 vaccines used in Sweden			Target population Age			
Vaccine	Strain	Use	Adults* ≥16 years	≥12 years	≥5 years	≥6 months
Comirnaty^®^ Pfizer/BioNTech BNT162b2 mRNA	Original strain (OS) Wuhan	Primary vaccination dose I + II	21 Dec 2020	28 May 2021	25 Nov 2021	19 Oct 2022
	OS	Booster dose III	24 Feb 2022	24 Feb 2022	15 Sep 2022	19 Oct 2022
	OS + Omicron BA.1	Booster dose III	01 Sep 2022	01 Sep 2022	-	-
	Omicron BA.4-5	Booster dose III	12 Sep 2022	12 Sep 2022	-	-
	Omicron BA.4-5	Single dose**	22 Jun 2023	22 Jun 2023	22 Jun 2023	22 Jun 2023
	Omicron XBB.1.5	Single dose**	30 Aug 2023	30 Aug 2023	30 Aug 2023	30 Aug 2023
	Omicron JN.1	Single dose**	27 Jun 2024	27 Jun 2024	27 Jun 2024	27 Jun 2024
	Omicron KP.2	Single dose**	20 Sep 2024	20 Sep 2024 ≥18 years	20 Sep 2024	20 Sep 2024
Spikevax^®^ Moderna mRNA-127	Original strain (OS) Wuhan	Primary vaccination	06 Jan 2021	23 Jul 2021	24 Feb 2022 (≥6 years)	19 Oct 2022
	OS	Booster dose III	25 Oct 2021	21 Jul 2022	-	-
	OS + Omicron BA.1	Booster dose III	01 Sep 2022	01 Sep 2022	16 Dec 2022 (≥5 years)	-
	Omicron BA.4-5	Booster dose III	19 Oct 2022	19 Oct 2022	26 Apr 2023	-
	Omicron BA.4-5	Single dose***	20 Jul 2023	20 Jul 2023	20 Jul 2023	20 Jul 2023
	Omicron XBB.1.5	Single dose***	14 Sep 2023	14 Sep 2023	14 Sep 2023	14 Sep 2023
	Omicron JN.1	Single dose***	10 Sep 2024 ≥18 years	10 Sep 2024	10 Sep 2024	10 Sep 2024
Vaxzevria^®^ AstraZeneca ChAdOx1 nCoV-19 Adenoviral vector	Original strain (OS) Wuhan	Primary vaccination	29 Jan 2021	-	-	-
	OS	Booster dose III	29 Jan 2021 ≥18 years	-	-	-
Nuvaxovid^®^ Novavax NVX-CoV2373 Protein	Original strain (OS) Wuhan	Primary vaccination	20 Dec 2021	23 Jun 2022	-	-
	OS	Booster dose III	01 Sep 2022	12 Oct 2023	-	-
	Omicron XBB.1.5	Single dose**	31 Oct 2023	31 Oct 2023	-	-
	Omicron JN.1	Single dose**	18 Oct 2024	18 Oct 2024	-	-

Footnotes: For specific details and references, see Appendix Table 1.

* Adults, that is, Comirnaty was first approved or ages ≥16 and the other three for ages ≥18 years.

** From 22 June 2023 the wording ‘a single dose’ is used in posology instead of ‘primary vaccination’ or ‘booster’ dose, that is, posology for dose IV and beyond.

*** Two doses primary vaccination and single doses.

From 22 February 2022 Comirnaty and Spikevax were recommended for use in pregnancy. For specific details, please see Appendix Table 2.

### EMA marketing authorisations

The four vaccines that were used in Sweden are listen in Table A1. During the vaccination campaign, changes in viral biology occurred and mRNA-vaccines were updated with respect to new variants discovered. The Omicron BA.1 variants of the mRNA vaccines were approved in September 2021 shortly followed by the BA.4-5 variant. Later an XBB.1.5 variant was approved in August 2023, a JN.1 variant in June 2024 and a KP.2 variant in September 2024. Previous vaccine variants production ceased with time and time points of approvals and of variations to the indications of the vaccines are described chronologically in the Appendix Table A3 below, including references to the EMA-website. Later, as an alternative to mRNA-vaccine, the protein-based vaccine Bimervax (Selvacovatein, Hipra Human Health) was authorised as an XBB.1 antigene-variant however, relatively late when the Omicron variants JN.1 and KP.2 of SARS-CoV-2 were already the dominating among circulating variants.

**Table A2 A-B:** Overview of safety events communicated via a Direct health care professional communication for Covid-19 vaccines in the EU in 2020-21 in panel A and updates on information of importance for vaccination recommendations issued in 2022 in panel B.

Panel A Vaccine	Safety concern	Date of EMAs first communications	Updates of product information recommended
Vaxzevria	Thrombocytopenia and coagulation disorders (thrombosis with thrombocytopenia syndrome (TTS)	10 Mar 2021	18 Mar 2021 07 Apr 2021
	DHPC Vaxevria	24 Mar 2021	
Comirnaty Spikevax	Myocarditis and pericarditis	07 May 2021	09 Jul 2021 03 Dec 2021
	DHPC Comirnaty and Spikevax	19 Jul 2021	
Vaxzevria	Capillary leak syndrome	11 Jun 2021	11 Jun 2021
Vaxzevria	Thrombocytopenia	10 Oct 2021	01 Oct 2021
**Panel B**			
Vaccine	From January 2022, safety updates are reported combined for all COVID-19-vaccines.		
Comirnat Spikevax	Can be used in pregnancy and during breast feeding (SmPC-update)	18 Jan 2022	22 Feb 2022

SmPC: Summary of Product Characteristics. From 22 February 2022 Comirnaty and Spikevax were recommended for use in pregnancy. For specific details, please see Appendix Table 2.

### EMA Pharmacovigilance activities

A risk management plan is part of a marketing authorisation of all new medicinal products including vaccines, and describes the summary of safety concerns, pharmacovigilance activities to be undertaken by a marketing authorisation holder and agreed risk minimisation measures. Pharmacovigilance surveillance is also performed by EMA and national competent authorities. Assessments of these activities were subsequently discussed in the Pharmacovigilance Risk Assessment Committee (PRAC) and risk communication activities were coordinated. For risks deemed as especially important, the latter included dissemination of a direct health care professional communication (DHPC), to relevant target groups of health care professionals, Appendix Table 2A–B. A DHPC is a formal communication sent by marketing authorisation holders to healthcare professionals. Its primary purpose is to inform about important new safety information regarding a medicine and any necessary actions needed to be taken.

In the Appendix Table 4A–B dates when these ADRs were identified and communicated as DHPCs are presented with links to the EMA-website.

### Detection of circulating COVID-19 variants continued

During the early months of the pandemic, testing was scarce because of very limited testing capacity available. From mid-March 2020, only hospitalised patients were tested. With time the capacity increased and testing was freely available from 01 August 2020 to 28 February 2022. In addition, self-tests (antigen tests) were developed and sold over the counter at pharmacies in Sweden. Results from these were not reported to any registers. Number of tests performed varied over time possibly because of several factors including availability of tests, virus spread in society and media attention. PCR-positive samples were regularly sequenced and phylogenetically analysed for follow-up on development of new virus variants.

A marked peak of positive tests was observed early in 2022, during the height of the presence of an omicron virus variant with high transmissibility coinciding with the last weeks of the period of freely available testing in Sweden.

### Detection of circulating COVID-19 variants

Over time, changes in viral biology occurred, resulting in the discovery of new virus genome mutations, and consequently different virus variants were dominating in occurrence in the population at different timepoints. In Figure 3, proportions of sequenced strains of SARS-CoV-2 in positive tests over time from 2020 to 2022, that is, alpha, beta, delta and omicron are presented. Later, the pattern of different omicron variants is more complex (data not shown). A continuous monitoring of variants of concern is performed by the European Centre of Disease Prevention and Control and presented in monthly updated listings.

**Table A3:** Approved COVID-19 vaccines in the EU up to 31^st^ December 2024.

Vaccine Posology	Target population (age)				Strain	Date, Reference
Comirnaty mRNA	≥16 years	≥12 years	≥5 years	≥6 months		
Primary course Dose I + II	X				Original (Wuhan-Hu-1) 30 µg	21 Dec 2020 (A)
Primary course Dose I + II		X			Original 30 µg	28 May 2021 (B)
Third primary dose in severely immune-compromised individuals.	X	X			Original 30 µg	04 Oct 2021 (C)
Primary course Dose I + II			X 5–11 years		Original 10 µg	25 Nov 2021 (D)
Booster Dose III For individuals who have received a primary course.	X	X			Original 30 µg	24 Feb 2022 (E)
Booster For individuals who had previously received at least a primary course.	X	X			Omicron BA.1 + Original 15+15 µg	01 Sep 2022 (F)
Booster For individuals who had previously received at least a primary course	X	X			Omicron BA.4-5 + Original 15+15 µg	12 Sep 2022 (G)
Booster Dose III For individuals who have received a primary course.			X 5–11 years		Original 10 µg	15 Sep 2022 (H)
Primary course Dose I+II+III				X 6 months – <5 years	Original 3 µg	19 Oct 2022 (I)
Single dose	X	X	X	X (3 doses primary course and single dose)	Omicron BA.4-5 + Original 15+15 µg (≥12 years) 5+5 µg (5–11 years) 1.5 + 1.5 (6 months – <5 years)	22 Jun 2023 (J)
Single dose	X	X	X	X (3 doses primary course and single dose)	Omicron XBB.1.5 30 µg (≥12 years) 10 µg (5–11 years) 3 µg (6 months – <5 years)	30 Aug 2023 (K)
Single dose	X	X	X	X (3 doses primary course and single dose)	Omicron JN.1 30 µg (≥12 years) 10 µg (5–11 years) 3 µg (6 months – <5 years)	27 Jun 2024 (L)
Single dose	X	X	X	X (3 doses primary course and single dose)	Omicron KP.2 30 µg (≥12 years) 10 µg (5–11 years) 3 µg (6 months – <5 years)	19 Sep 2024 (M)
**Spikevax** mRNA	≥18 years	≥12 years	≥6 years	≥6 months		
Primary course Dose I+II	X (e.g. ≥18)				Original (Wuhan-Hu-1) 100 µg	06 Jan 2021 (N)
Primary course Dose I+II		X			Original 100 µg	23 Jul 2021 (O)
Third primary dose in severely immune-compromised	X	X			Original 100 µg	04 Oct 2021 (C)
Booster Dose III For individuals who have received a primary course against Covid-19	X				Original 50 µg	25 Oct 2021 (P)
Primary course Dose I+II Third primary dose in severely immuno-compromised			X 6–11 years		Original 50 µg	24 Feb 2022 (Q)
Booster Dose III For individuals who have received a primary course against Covid-19		X			Original 50 µg	21 Jul 2022 (R)
Booster For individuals who had previously received at least a primary course against Covid-19	X	X			Omicron BA.1 + Original 25+25 µg	01 Sep 2022 (F)
Booster For individuals who had previously received at least a primary course against Covid-19	X	X			Omicron BA.4-5 + Original 25+25 µg	19 Oct 2022 (S)
Primary course Dose I + II				X 6 months – <6 years	Original 25 µg	19 Oct 2022 (T)
Booster For individuals who had previously received at least a primary course against Covid-19			X (5–11 years)		Omicron BA.1 + Original 12.5 + 12.5 µg	16 Dec 2022 (U)
Booster For individuals who had previously received at least a primary course against Covid-19			X (5–11 years)		Omicron BA.4-5 + Original 12.5 + 12.5 µg	26 Apr 2023 (V)
Single dose	X	X	X	X 6 months – <5 years (2 doses primary course and single dose)	OmicronBA.4-5 + Original 25+25 µg (≥12 years) 12.5 + 12.5 µg (6 months-11 years)	20 Jul 2023 (X)
Single dose	X	X	X	X (2 doses primary course and single dose)	Omicron XBB.1.5 50 µg (≥12 years) 25 µg (6 months-11 years)	14 Sep 2023 (Y)
Single dose	X	X	X	X (2 doses primary course and single dose)	Omicron JN.1 50 µg (≥12 years) 25 µg (6 months-11 years)	9 Sep 2024 (Z)
**Vaxzevria** Adenoviral vector	≥18 years					
Primary course Dose I + II	X				Original (Wuhan-Hu-1)	29 Jan 2021 (AA)
Booster Dose III	X				Original	20 May 2022 (AB)
Withdrawal of the marketing authorisation in the EU upon request from the Marketing Authorisation Holder					Original	27 Mar 2024 (AC)
**JCovden** Adenoviral vector	≥18 years					
Primary course Dose I	X				Original (Wuhan-Hu-1)	11 Mar 2021 (AD)
Booster Dose II	X				Original	14 Dec 2021 (AE)
Booster Dose III	X				Original	10 Nov 2022 (AF)
Withdrawal of the marketing authorisation in the EU upon request from the MAH					Original	26 Jul 2024 (AG)
**Nuvaxovid** Protein	≥18 years	≥12 years				
Primary course Dose I+II	X				Original (Wuhan-Hu-1)	20 Dec 2021 (AH)
Primary course Dose I+II		X			Original	23 Jun 2022 (AI)
Booster Dose III For individuals who have received a primary course	X				Original	01 Sep 2022 (AJ)
Booster Dose III For individuals who have received a primary course		X			Original	12 Oct 2023 (AK)
Single dose	X	X			Omicron XBB.1.5	31 Oct 2023 (AL)
Single dose	X	X			Omicron JN.1	7 Oct 2024 (AM)
**Bimervax** Protein	≥16 years					
Booster Dose III For individuals who have received a primary course of mRNA vaccine against Covid-19	X				Alpha + Beta (B.1.1.7+ B.1.351)	30 Mar 2023 (AN)
Booster for individuals who have received a previous booster with Bimervax	X				Alpha + Beta (B.1.1.7+ B.1.351)	22 Feb 2024 (AO)
Single dose	X				Omicron XBB.1.16	17 Oct 2024 (AP)
**Valneva** inactivated, adjuvanted	≥18–50 years					
Primary course Dose I + II	X				Wuhan-Hu-1	24 Jun 2022 (AQ)
Booster Dose III For individuals who have received a primary course of mRNA or adenoviral vector vaccine against Covid-19	X				Wuhan-Hu-1	23 Feb 2023 (AR)
Withdrawal of the marketing authorisation in the EU upon request from the Marketing Authorisation Holder					Wuhan-Hu-1	12 Oct 2023 (AS)
**VidPrevtyn Beta** recombinant, adjuvanted	≥18 years					
Booster Dose III For individuals who have received a primary course of mRNA or adenoviral vector vaccine	X				Beta (B.1.351)	10 Nov 2022 (AT)
Withdrawal of the marketing authorisation in the EU upon request from the MAH					Beta (B.1.351)	18 Mar 2024 (AU)

From 22 February 2022 Comirnaty and Spikevax were recommended for use in pregnancy. The Pharmacovigilance and Risk Assessment Committee (PRAC) provided advice to the Committee for Medicinal Products for Human Use (CHMP) which recommended updating the product information of the mRNA vaccines Comirnaty and Spikevax regarding their use in pregnancy and breastfeeding. For details, please see Appendix table A2, reference P.

From 22 June 2023 the wording ‘a single dose’ is used in posology instead of ‘primary vaccination’ or ‘booster’ dose.

**References list Table A3:** Procedure links to EMA documentation.

Comirnaty	Comirnaty | European Medicines Agency (europa.eu)
A	EMA recommends first COVID-19 vaccine for authorisation in the EU | European Medicines Agency (europa.eu)
B	First COVID-19 vaccine approved for children aged 12 to 15 in EU | European Medicines Agency (europa.eu)
C	Comirnaty and Spikevax: EMA recommendations on extra doses and boosters | European Medicines Agency (europa.eu)
D	Comirnaty COVID-19 vaccine: EMA recommends approval for children aged 5 to 11 | European Medicines Agency (europa.eu)
E	EMA recommends authorisation of booster doses of Comirnaty from 12 years of age | European Medicines Agency (europa.eu)
F	First adapted COVID-19 booster vaccines recommended for approval in the EU | European Medicines Agency (europa.eu)
G	Adapted vaccine targeting BA.4 and BA.5 Omicron variants and original SARS-CoV-2 recommended for approval | European Medicines Agency (europa.eu)
H	EMEA/H/C/005735/II/0129 Comirnaty, INN-tozinameran, tozinameran/riltozinameran (europa.eu)
I	EMA recommends approval of Comirnaty and Spikevax COVID-19 vaccines for children from 6 months of age | European Medicines Agency (europa.eu)
J	EMEA/H/C/005735/II/0177/G Comirnaty | European Medicines Agency (EMA) (europa.eu)
K	Comirnaty: EMA recommends approval of adapted COVID-19 vaccine targeting Omicron XBB.1.5 | European Medicines Agency (europa.eu)
L	EMEA/H/C/005735/II/0216/G Comirnaty | European Medicines Agency (EMA) (europa.eu)
M	Comirnaty, INN-tozinameran, tozinameran/riltozinameran, tozinameran/famtozinameran
**Spikevax**	Spikevax (previously COVID-19 Vaccine Moderna) | European Medicines Agency (europa.eu)
N	EMA recommends COVID-19 Vaccine Moderna for authorisation in the EU | European Medicines Agency (europa.eu)
O	COVID-19 vaccine Spikevax approved for children aged 12 to 17 in EU | European Medicines Agency (europa.eu)
P	Spikevax: EMA recommendation on booster | European Medicines Agency (europa.eu)
Q	EMA recommends approval of Spikevax for children aged 6 to 11 | European Medicines Agency (europa.eu)
R	EMEA/H/C/005791/II/0057 Spikevax (previously COVID-19 Vaccine Moderna) | European Medicines Agency (EMA) (europa.eu)
S	EMA recommends approval of second adapted Spikevax vaccine | European Medicines Agency (europa.eu)
T	EMA recommends approval of Comirnaty and Spikevax COVID-19 vaccines for children from 6 months of age | European Medicines Agency (europa.eu)
U	Meeting highlights from the Committee for Medicinal Products for Human Use (CHMP) 12–15 December 2022 | European Medicines Agency (europa.eu)
V	Meeting highlights from the Committee for Medicinal Products for Human Use (CHMP) 24–26 April 2023 | European Medicines Agency (europa.eu)
X	EMEA/H/C/005791/II/104/G Spikevax (previously COVID-19 Vaccine Moderna) | European Medicines Agency (EMA) (europa.eu)
Y	Spikevax: EMA recommends approval of adapted COVID-19 vaccine targeting Omicron XBB.1.5 | European Medicines Agency (europa.eu)
Z	Meeting highlights from the Committee for Medicinal Products for Human Use (CHMP) 16–19 September 2024 | European Medicines Agency (EMA)
**Vaxzevria**	Vaxzevria (previously COVID-19 Vaccine AstraZeneca) | European Medicines Agency (europa.eu)
AA	EMA recommends COVID-19 Vaccine AstraZeneca for authorisation in the EU | European Medicines Agency (europa.eu)
AB	Meeting highlights from the Committee for Medicinal Products for Human Use (CHMP) 16–19 May 2022 | European Medicines Agency (europa.eu)
AC	‘Vaxzevria (previously COVID-19 Vaccine AstraZeneca), INN-COVID-19-Vaccine-(ChAdOx1-S-[recombinant])’ (europa.eu)
**JCovden**	Jcovden (previously COVID-19 Vaccine Janssen) | European Medicines Agency (europa.eu)
AD	EMA recommends COVID-19 Vaccine Janssen for authorisation in the EU | European Medicines Agency (europa.eu)
AE	COVID-19 Vaccine Janssen: EMA recommendation on booster dose | European Medicines Agency (europa.eu)
AF	EMEA/H/C/005737/II/0053/G Draft CHMP Agenda 07–10 November 2022 Corr.1 (europa.eu)
AG	JCOVDEN, INN-Ad26.COV2-S, recombinant (europa.eu)
**Nuvaxovid**	Nuvaxovid | European Medicines Agency (europa.eu)
AH	EMA recommends Nuvaxovid for authorisation in the EU | European Medicines Agency (europa.eu)
AI	EMA recommends authorisation of Nuvaxovid for adolescents aged 12 to 17 | European Medicines Agency (europa.eu)
AJ	EMEA/H/C/005808/II/0014 Nuvaxovid | European Medicines Agency (EMA) (europa.eu)
AK	EMEA/H/C/005808/II/0045 Nuvaxovid, INN-COVID-19 Vaccine (recombinant, adjuvanted) (europa.eu)
AL	EMA recommends approval of adapted Nuvaxovid COVID-19 vaccine targeting Omicron XBB.1.5 | European Medicines Agency (europa.eu)
AM	Meeting highlights from the Committee for Medicinal Products for Human Use (CHMP) 14–17 October 2024 | European Medicines Agency (EMA)
**Bimervax**	Bimervax | European Medicines Agency (EMA) (europa.eu)
AN	EMEA/H/C/006058/0000 Bimervax, INN-RBD fusion heterodimer (europa.eu) CHMP Minutes 27–30 March 2023 (europa.eu)
AO	EMEA/H/C/006058/II/0004 CHMP Minutes 19–22 February 2024 (europa.eu)
AP	Bimervax INN-RBD fusion heterodimer
**Valneva**	Union Register of medicinal products – Public health – European Commission (europa.eu)
AQ	EMA recommends Valneva’s COVID-19 vaccine for authorisation in the EU | European Medicines Agency (EMA) (europa.eu)
AR	CHMP Minutes 20–23 February 2023 (europa.eu)
AS	EMEA/H/C/006019/II/0004 ‘COVID-19 Vaccine (inactivated, adjuvanted) Valneva suspension for injection, COVID-19 vaccine (inactivated, adjuvanted, adsorbed)’ (europa.eu)
**VidPrevtyn Beta**	VidPrevtyn Beta | European Medicines Agency (EMA) (europa.eu)
AT	EMEA/H/C/005754/0000 VidPrevtyn Beta, INN-COVID-19-Vaccine-(recombinant,-adjuvanted) (europa.eu)
AU	VidPrevtyn Beta, COVID-19 vaccine (recombinant, adjuvanted) (europa.eu)
GENERAL COVID-19 Vaccine Information	COVID-19 medicines | European Medicines Agency (EMA) COVID-19: latest updates (archive) | European Medicines Agency (EMA)

Footnote: For further details, please see the European Commission Public Health – Union Register of medicinal products listing of COVID-19 vaccines: Union Register of medicinal products – Public health – European Commission (europa.eu)

**Table A4 A-B:** Overview of safety events communicated via a Direct Health Care Professional communication for Covid-19 vaccines in the EU in 2020–2021 in panel A and updates on important information for vaccination recommendations in 2020–2024 in panel B.

Panel A Vaccine	Safety concern	Date of EMAs first and subsequent communications	Updates of product information recommended
Vaxzevria	Thrombocytopenia and coagulation disorders (thrombosis with thrombocytopenia syndrome (TTS)	10 March 2021 (A) 11 Mar 16 Mar 18 Mar 25 Mar 31 Mar 07 Apr 14 Apr 21 May 07 Jun	18 Mar 2021 (B) 07 Apr 2021 (C)
	DHPC Vaxzevria	24 Mar 2021 (D)	
Jcovden	Thrombocytopenia and coagulation disorders (thrombosis with thrombocyto-penia syndrome (TTS)	14 April 2021 (E) 20 Apr 07 May 07 Jun	20 Apr F) 07 May (G)
	DHPC JCovden	26 Apr 2021 (H)	
Comirnaty Spikevax	Myocarditis and pericarditis	7 May 2021 (I) 11 Jun 09 July 29 Oct 03 Dec	09 Jul 2021 (J) 03 Dec 2021 (K)
	DHPC Comirnaty and Spikevax	19 Jul 2021 (L)	
Vaxzevria	Capillary leak syndrome	11 Jun 2021 (M)	11 Jun 2021
Jcovden	Capillary leak syndrome	09 Jul 2021(N)	09 Jul 2021
Jcovden	Immune thrombocytopenia	05 Aug 2021(O)	05 Aug 2021
Jcovden	Venous thromboembolism	03 Sept 2021 (P) 01 Oct 2021	01 Oct 2021 (Q)
Vaxzevria	Thrombocytopenia	01 Oct 2021 (R)	01 Oct 2021
**Panel B** Vaccine	From January 2022 are safety updates reported combined for all COVID-19-vaccines.		
mRNA-vaccines	Can be used in pregnancy and breast feeding (SPC-update)	18 Jan 2022	22 Feb 2022 (S)
mRNA-vaccines	Heavy menstrual bleeding (SPC-update)	10 Nov 2022	10 Nov 2022 (T)
mRNA-vaccines	Post menopausal bleeding not linked to vaccines	08 Mar 2024	08 Mar 2024 (U)

SmPC: Summary of Product Characteristics. Direct Health Care Professional communication.

Footnote: From 22 February 2022 Comirnaty and Spikevax were recommended for use in pregnancy. The Pharmacovigilance and Risk Assessment Committee (PRAC) provided advice to the Committee for Medicinal Products for Human Use (CHMP) which recommended updating the product information of the mRNA vaccines Comirnaty and Spikevax regarding their use in pregnancy and breastfeeding.

**References list Table A4 A–B:** European Medicines Agency Procedure links to documentation.

Ref	Link to document	Adverse drug reaction	Vaccine
A	COVID-19 Vaccine AstraZeneca: PRAC preliminary view suggests no specific issue with batch used in Austria | European Medicines Agency (europa.eu)	Thrombocytopenia and coagulation disorders (thrombosis with thrombocytopenia syndrome (TTS)	Vaxzevria
B	COVID-19 Vaccine AstraZeneca: benefits still outweigh the risks despite possible link to rare blood clots with low blood platelets | European Medicines Agency (europa.eu)		
C	AstraZeneca’s COVID-19 vaccine: EMA finds possible link to very rare cases of unusual blood clots with low blood platelets | European Medicines Agency (europa.eu)		
D	DHPC: COVID-19 Vaccine AstraZeneca: Risk of thrombocytopenia and coagulation disorders		
E	COVID-19 Vaccine Janssen: assessment of very rare cases of unusual blood clots with low platelets continues | European Medicines Agency (europa.eu)		Jcovden
F	COVID-19 Vaccine Janssen: EMA finds possible link to very rare cases of unusual blood clots with low blood platelets | European Medicines Agency (europa.eu)		
G	Meeting highlights from the Pharmacovigilance Risk Assessment Committee (PRAC) 3–6 May 2021 | European Medicines Agency (europa.eu)		
H	COVID-19 Vaccine Janssen: link between the vaccine and the occurrence of thrombosis in combination with thrombocytopenia		
I	Meeting highlights from the Pharmacovigilance Risk Assessment Committee (PRAC) 3–6 May 2021 | European Medicines Agency (europa.eu)	Myocarditis and pericarditis	Comirnaty Spikevax
J	Comirnaty and Spikevax: possible link to very rare cases of myocarditis and pericarditis | European Medicines Agency (europa.eu)		
K	Meeting highlights from the Pharmacovigilance Risk Assessment Committee (PRAC) 29 November – 2 December 2021 | European Medicines Agency (europa.eu)		
L	COVID-19 mRNA Vaccines Comirnaty and Spikevax		
M	Vaxzevria: EMA advises against use in people with history of capillary leak syndrome | European Medicines Agency (europa.eu)	Capillary leak syndrome	Vaxzevria
N	EMA advises against use of COVID-19 Vaccine Janssen in people with history of capillary leak syndrome | European Medicines Agency (europa.eu)		Jcovden
O	Meeting highlights from the Pharmacovigilance Risk Assessment Committee (PRAC) 5 August 2021 | European Medicines Agency (europa.eu)	Immune thrombocytopenia	Jcovden
P	Link to original EMA-document broken. (Reference is given to EMA overview: Jcovden (previously COVID-19 Vaccine Janssen) | European Medicines Agency (EMA))	Venous thromboembolism	Jcovden
Q	Meeting highlights from the Pharmacovigilance Risk Assessment Committee (PRAC) 27–30 September 2021 | European Medicines Agency (europa.eu)		
R	Meeting highlights from the Pharmacovigilance Risk Assessment Committee (PRAC) 27–30 September 2021 | European Medicines Agency (europa.eu)	Thrombocytopenia	Vaxzevria
S	COVID-19 Vaccines Safety Update February 2022 (europa.eu)	Usage in pregnancy and breastfeeding	mRNA-vaccines
T	COVID-19 Vaccines Safety Update November 2022 (europa.eu)	Heavy menstrual bleeding	mRNA-vaccines
U	Meeting highlights from the Pharmacovigilance Risk Assessment Committee (PRAC) 4–7 March 2024 | European Medicines Agency (europa.eu)	Post menopausal bleeding not linked to vaccines	mRNA-vaccines

**Table A5:** Recommendations issued by the Swedish Public Health Agency on the phased vaccine plan for use of COVID-19 vaccines in Sweden.

Date communicated	Vaccine usage	Target population recommendations	Vaccine/strain
27 Dec 2020	Primary vaccine Doses I + II	First Phase: Nursing Home Residents (NHR)	Comirnaty
02 Feb 2021		Health Care Professionals (HCP) 18–64 years of age	Vaxzevria
04 Feb 2021		Second Phase 65 years of age and above	Comirnaty Spikevax
04 Feb 2021		Third Phase 60–64 years of age and younger with certain diseases with increased risk of severe COVID-19^a^.	Vaxzevria
04 Mar 2021		All approved vaccines available in Sweden may be used from 18 years of age and above	Comirnaty Spikevax Vaxzevria
11 Mar 2021		A fourth vaccine (single dose) from 18 years of age and above will be available in April	Jcovden
16 Mar 2021	Restriction	Vaxzevria usage paused because of suspected ADR; TTS	Vaxzevria
19 Mar 2021	Priority	Higher ages are to be prioritised, and dose number two may be delayed	
25 Mar 2021	Priority and restriction	Vaxzevria may be used in 65 years of age and above but is still restricted for usage in 64 years and younger	Vaxzevria
14 Apr 2021	Restriction	Jcovden on hold, not to be used until EMAs risk assessment on possible ADR; TTS is finalised	Jcovden
20 Apr 2021	Switch	mRNA vaccine recommended for second dose after first dose of Vaxzevria	Comirnaty Spikevax
23 Apr 2021	Restriction	Jcovden on hold continued, not to be used in any age group	Jcovden
26 Apr 2021		Vaxzevria may be used as second dose after first dose of Vaxzevria if individuals so prefer	Vaxzevria
27 Apr 2021	Priority	Pregnant women with complicating diseases with increased risk of severe COVID-19 may be vaccinated (mRNA) in the third phase	Comirnaty Spikevax
25 May 2021	Priority	Pregnant women may be vaccinated (mRNA) in the fourth phase in the same priority order as anybody else	Comirnaty Spikevax
22 Jun 2021	Priority	All at age 16 and above are recommended vaccination	Comirnaty
14 Jul 2021	Priority	All born in 2005 or earlier may be vaccinated from week 32/2021 after the fourth phase of vaccinations	Comirnaty Spikevax
23 Jul 2021	Priority	All at age 16 and above are recommended vaccination	Spikevax
23 Jul 2021	Restriction	Vaxzevria use is recommended to gradually end, and mRNA vaccines are to be used onwards	Comirnaty Spikevax Vaxzevria
26 Aug 2021	Booster - Dose III	Individuals with severely weakened immune system age 18 and above from 01 Sept 2021 onwards	Comirnaty Spikevax
16 Sep 2021	Primary vaccination	General vaccination from age 12 and above recommended	Comirnaty Spikevax
28 Sep 2021	Booster - Dose III	Individual with increased risk of severe COVID-19 and NHR, HCP, age 80 and above	Comirnaty Spikevax
04 Oct 2021	Restriction	Only Comirnaty and not Moderna are to be used in ages 12 – 15 years	Comirnaty
06 Oct 2021	Restriction	Spikevax not to be used under ages of 30 years (born 1991 and later) because of increased risk of myocarditis and pericarditis	Spikevax
21 Oct 2021	Restriction	Comirnaty to be used as second dose if first dose were Spikevax in ages under 30 years.	Comirnaty
27 Oct 2021	Booster - Dose III	All above age 65 and HCP, 6 months after dose II	Comirnaty Spikevax
10 Nov 2021	Booster - Dose III	All above age 65 and HCP, 5 months after dose II	Comirnaty Spikevax
24 Nov 2021	Priority Dose III	Comirnaty to all from age 18 and Spikevax from age 30, in sequence of highest needs first, for booster dose III.	Comirnaty Spikevax
21 Dec 2021	Booster -dose III	In age 5 and above with high risk of severe airway infections	Comirnaty
12 Jan 2022	Booster -dose III	All booster doses should be given after 5 months after previous dose	Comirnaty Spikevax
21 Jan 2022	Booster -dose III	Individuals with severely weakened immune system should have a booster dose within 2–3 months after primary vaccinations	Comirnaty Spikevax
27 Jan 2022	General vaccination	General vaccination is not recommended for children aged 5–11 years during first half of 2022	Comirnaty
31 Jan 2022	Booster - dose III	All booster doses should be given at least 3 months after previous dose	Comirnaty Spikevax
14 Feb 2022	Booster -dose IV	NHR and aged 80 and above, booster dose within 4 months from previous dose	Comirnaty Spikevax
04 Apr 2022	Booster -dose IV	Booster doses for all 65 years of age and above	Comirnaty Spikevax
24 May 2022	Booster -dose V	From 01 Sep 2022 a booster dose to all with increased risk for serious COVID-19, including pregnant and age 65 years and above	Comirnaty Spikevax
30 Sep 2022	General vaccination	General vaccination recommendation for healthy children, ages 12–17 years to end by 31 Oct 2022	Comirnaty Spikevax
02 Nov 2022	Restriction	Nuvaxovid not to be given in ages 30 years and below because of possible risk of myocarditis.	Nuvaxovid
22 Dec 2022	Booster doses	From 01 Mar 2023 are two booster doses per year in ages above 80, NHR. One booster dose per year for ages 65–79 years	Comirnaty Spikevax
07 Feb 2023		From 01 Mar 2023 to 29 Feb 2024, a booster for ages 50–64 for the winter season 2023/24.	Comirnaty Spikevax
10 May 2023	WHO Announcement	WHO have decided after assessment that COVID-19 no longer is to be deemed as an international threat to public health (PHEIC) from 05 May 2023	Comirnaty Spikevax
19 Mar 2024	Booster doses	The spring of 2024: A booster for those in ages above 80 and for those in ages above 65 in NHCR	Comirnaty Spikevax
27 June 2024 Updated: 29 August 2024	Booster dose	From 01 Sep 2024, a booster for all persons aged 65 and above and for defined risk groups; 18 years and above for the winter season 2024/25. Persons aged 80 and above or 65 and above if using HSCH or being NHCR an additional booster during the spring of 2025. Persons with severe immune deficiencies may need extra booster dosing	Comirnaty Spikevax

ADR: Adverse Drug reaction; HCP: Health Care Professionals; HSHC: Home Service Health Care; NHCR: Nursing Home Care Residents; TTS: Thrombosis with thrombocytopenia syndrome.

Footnote: Risk groups defined in issued recommendations were: Hypertension, Diabetes Mellitus, Obesity, Chronic obstructive pulmonary disease and severe asthma.

### References Table A5 (In Swedish)

Rekommendationer om vaccination mot covid-19 (folkhalsomyndigheten.se)

Nationella vaccinationsregistret – Folkhälsomyndigheten

### Recommendations for use in children

As of February 2021, the PHA had not recommended COVID-19 vaccination to children under 18 unless the child belonged to a high-risk group, Appendix Table A5. The recommendation was changed in June 2021 to offer everyone at 16 years of age and above COVID-19 vaccination.

From 04 October 2021, Comirnaty, but not Spikevax, were to be used for those 12 to 15 years old. On 06 October 2021 came further restrictions on Spikevax. It was not to be used among those aged up to 30 years, because of increased risk of myocarditis and pericarditis.

On 27 January 2022, the PHA decided against recommending general vaccinations against COVID-19 for children, aged below 12, estimating that the benefits did not outweigh possible risks. Children in high-risk groups could, however, be vaccinated at their physician’s discretion. In October 2022, general vaccination for children below 18 years of age was ended.

Recommendations on use in children differed between the Nordic countries, with Denmark moving forward rather swiftly with recommendations for general vaccination of children of decreasing age but also being the first country to end the recommendation for children in April 2022. Norway implemented a one-dose primary vaccination strategy for most children as a mean to avoid side effects and as the immunological response in children was found to be very high.

**Table A6:** Intervals in days between COVID-19 vaccine doses administered in Sweden from 27 December 2020 to 31 December 2024

Dose sequences	*n*	Min	Q1	Median	Q3	Max
1 to 2	7,700,925	1	39	42	49	1,386
2 to 3	5,651,070	1	172	189	211	1,407
3 to 4	3,149,725	1	142	156	287	1,381
4 to 5	2,211,211	1	154	184	357	1,141
5 to 6	1,710,766	1	224	362	415	1,103
6 to 7	1,267,439	1	215	327	349	924
7 to 8	511,218	1	147	175	266	786
8 to 9	242,005	1	183	196	210	602
9 to 10	4,364	1	164	189	205	482

*n* denotes number of persons vaccinated; Q1 and Q3 denotes inter quartile borders.
